# An IMU-Based Wearable System for Respiratory Rate Estimation in Static and Dynamic Conditions

**DOI:** 10.1007/s13239-023-00657-3

**Published:** 2023-02-27

**Authors:** Alessandra Angelucci, Andrea Aliverti

**Affiliations:** Dipartimento di Elettronica, Informazione e Bioingegneria, Politecnico di Milano, Piazza Leonardo Da Vinci 32, 20133 Milan, Italy

**Keywords:** e-Health, Human activity recognition, Respiratory monitoring, Internet of medical things, Telemedicine

## Abstract

**Purpose:**

Breathing parameters change with activity and posture, but currently available solutions can perform measurements only during static conditions.

**Methods:**

This article presents an innovative wearable sensor system constituted by three inertial measurement units to simultaneously estimate respiratory rate (RR) in static and dynamic conditions and perform human activity recognition (HAR) with the same sensing principle. Two units are aimed at detecting chest wall breathing-related movements (one on the thorax, one on the abdomen); the third is on the lower back. All units compute the quaternions describing the subject’s movement and send data continuously with the ANT transmission protocol to an app. The 20 healthy subjects involved in the research (9 men, 11 women) were between 23 and 54 years old, with mean age 26.8, mean height 172.5 cm and mean weight 66.9 kg. Data from these subjects during different postures or activities were collected and analyzed to extract RR.

**Results:**

Statistically significant differences between dynamic activities (“walking slow”, “walking fast”, “running” and “cycling”) and static postures were detected (*p* < 0.05), confirming the obtained measurements are in line with physiology even during dynamic activities. Data from the reference unit only and from all three units were used as inputs to artificial intelligence methods for HAR. When the data from the reference unit were used, the Gated Recurrent Unit was the best performing method (97% accuracy). With three units, a 1D Convolutional Neural Network was the best performing (99% accuracy).

**Conclusion:**

Overall, the proposed solution shows it is possible to perform simultaneous HAR and RR measurements in static and dynamic conditions with the same sensor system.

## Introduction

A wearable device is a technology that can be worn, incorporated in clothes or as an accessory, and provides a series of signals and data regarding the subject’s status and health, as well as data regarding the surrounding environment.^1^ By means of wearables, it is possible to obtain physiological parameters without interfering with daily life activities, to reduce the obtrusiveness to a minimum and to enable a continuous and extended monitoring. Wearables find applications in several fields, including health, wellbeing, and fitness.^4^

Examples of parameters which can be measured are body and skin temperature, respiratory rate (RR), heart rate (HR) and pulse rate (PR), arterial blood pressure (ABP), blood glucose concentration, galvanic skin response (GSR) or electrodermal activity (EDA), peripheral capillary oxygen saturation (SpO_2_), photoplethysmogram (PPG), electrocardiogram (ECG), electroencephalogram (EEG), electromyogram (EMG),^18^ as well as parameters regarding the surrounding environment such as temperature, humidity, air pressure, and concentrations of various pollutants.^12^

Wearables can be integrated in telemonitoring system, which usually follow the so-called two-hop architecture.^24^ Its name derives from the fact that there are two steps of data transmission: the first from sensors to a gateway, with the communication performed by a sensor-manager link technology, and the second from the gateway to data management section, thanks to cellular link technologies such as Wi-Fi, 4G, and 5G.^5^ The networks that are obtained are called Wireless Body Area Networks, or WBANs.^1^

### Remote Monitoring of Respiratory Parameters

In the healthcare field, the opportunity to identify abnormalities in RR is fundamental to forecast cardiac arrest,^22^ exacerbations,^23^ admissions to the Intensive Care Unit, and other adverse clinical events.^39^

Despite the relevance of RR as prognostic factor, the current gold standard for measuring RR is the number of breaths performed in one minute, identified through auscultation or observation, which is not suitable for prolonged monitoring outside the clinical environment. An alternative to this is the employment of dedicated devices, but a limitation that is found in several studies is that physiological parameters are usually detected with spot measurements and when the subject is at rest, while it is known that physical activity has an influence on cardiorespiratory function.^21,33^

Systems for continuous monitoring of breathing can use wearables based on different technologies: PPG-derived signals (*e.g.*, in smartwatches^31^), respiratory inductance plethysmography (RIP),^41^ resistance-based sensors,^11,19,42^ capacitance-based sensors,^32^ inertial measurement units (IMUs),^16,42^ or fiber optic sensors.^29^ Some of these sensors can be embedded in garments.^8^ Although there are many proposed solutions, there is still a paucity of commercially available devices that are dedicated to respiratory parameters. The most commonly commercially available solutions are the smartwatches with PPG sensors, but the PPG signal is reliable for RR estimation only in resting conditions, due to the excessive motion artifacts present when subjects are performing dynamic activities. Considering the decreased intensity of PPG-derived respiratory signal with increasing RR, high RR is difficult to accurately detect from PPG signals, especially for values higher than 30 breaths per minute (bpm).^27^ Other technologies have different limitations. Most devices based on the movement of the chest wall and described in the literature only acquire the respiratory monitoring with one degree of freedom. However, it is known in the literature that different regions of the chest wall contribute to the breathing activity, and the level of contribution changes in different postures.^37^ In RIP, two bands are applied, one at the thoracic level and one at the abdominal level, but the slippage of bands can lead to inaccurate readings.^38^

The advantage of using wearable devices is that they are not cumbersome and can be used to monitor physiological parameters during daily life activities and outside of clinical settings. However, as respiratory parameters are known to change during different activities and in different postures,^20^ having a system that combines respiratory parameters in static and dynamic conditions and human activity recognition (HAR) would provide even more clinically relevant information.

### Activity Recognition Systems

The current wearable technologies that can be used to implement HAR can be sensor-based, vision-based, or radio-based. Sensor-based technologies are the ones that can be used without environmental constraints. Linear accelerations^13^ and angular velocities^26^ can be detected *via* micro-electro-mechanical systems (MEMS), which measure either capacity changes or the deflection of magnetically excited comb structures. Their use is based on demonstrated relationships between accelerometer output and energy expenditure in studies on gait analysis and ergonomics.^44^ Barometric pressure sensors, on the other hand, can be particularly useful in fall detection.^30^ Data obtained from the employed sensors can then be fed to an artificial intelligence algorithm based on machine or deep learning techniques.

### Aim of the Work

The present research work has three main goals: one is to present an advanced prototype suitable for continuous monitoring of respiratory parameters in static and dynamic conditions; another one is to exploit the possibility to perform HAR from the same raw sensor data used for respiratory monitoring; the third one is to use the knowledge on performed activity to fine-tune a RR estimation algorithm and thus improve accuracy. The first point was addressed by optimizing a previously validated IMU-based technology, while the second was performed with artificial intelligence methods. These points are addressed in "Materials and Methods" section. The third point is presented from the post-processing point of view in "Results" section and a possible workflow for future implementations is later discussed in "Discussion" section.

## Materials and Methods

### Dataset

The 20 healthy subjects involved in the research (9 men, 11 women) were between 23 and 54 years old at the time of the study, with mean age 26.8, mean height 172.5 cm and mean weight 66.9 kg. The experimentation was approved by the Ethical Committee of Politecnico di Milano (Protocol number: 20/2020) and all participants signed an informed consent.

**Figure 1:**
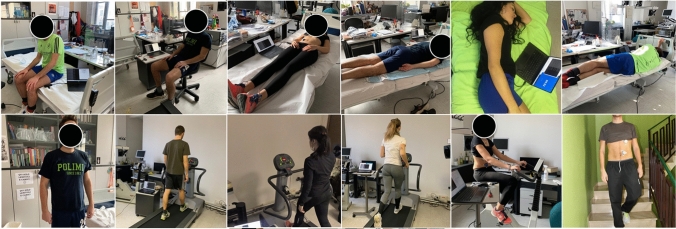
Healthy subjects involved in the experimental protocol while performing the various activities.

The protocol, shown in Fig. 1, included seven static postures (sitting with support, sitting without support, supine, prone, left decubitus, right decubitus, standing) and five dynamic activities (walking slow at 4 km/h, walking fast at 6 km/h, running, climbing up and down the stairs, cycling). Each activity lasted 5 min. The walking and running activities were performed on a treadmill, while the cycling activity was performed on an ergometer.

### Hardware, Firmware, and Data Transmission

The present work exploits a wearable respiratory Holter based on three Inertial Measurement Units (IMUs).^2^ Two sensor units are aimed at detecting chest wall breathing-related movements, one located on the thorax and the other on the abdomen; the last IMU is placed in a position not involved in respiratory motion but integral with body movement.^15,16^

Data from the 9-axis IMUs were previously validated to extract respiratory parameters in static conditions. An algorithm for the offline processing of the obtained data was developed and validated with Opto-Electronic Plethysmography (OEP) on healthy subjects.^17^ A comparison with OEP for the breathing frequency estimation demonstrated that the device based on the inertial measurement units (IMU-based device) provided optimal results in terms of mean absolute errors (< 2 breaths/min) and correlation (r > 0.963). However, raw data were never exploited in terms of HAR, which is of great interest in combination with respiratory parameters.

**Figure 2:**
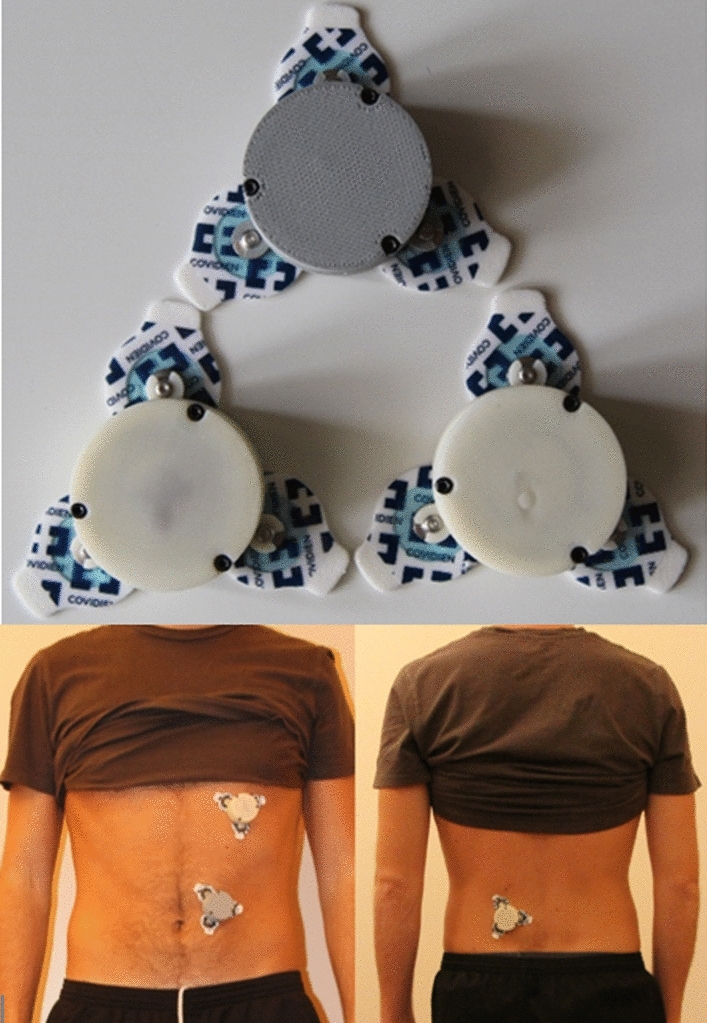
Three units composing the wearable system (top); thoracic and abdominal units worn by a male subject (bottom left); reference unit worn by a male subject and placed on the lower back (bottom right).

In this paper, the reference unit is placed on the lower back because its movement is integral with the one of the body trunk, but not involved in respiratory movement. The main components of each unit of the device are an inertial measurement unit (ICM-20948), and a microcontroller module with an integrated ANT transceiver (MDBT42Q, based on the microcontroller nRF52832). The device is attached to the skin of the subject with disposable ECG electrodes. The three units composing the wearable systems, once closed, are represented in Fig. 2 (top). In the same figure (bottom left), the thoracic and the abdominal unit are shown when a subject is wearing them. The thoracic unit is placed at the level of the abdominal rib cage, while the abdominal unit is placed next to the belly button. Fig. 2 also shows the reference unit placed on the lower back of the same subject (bottom right).

Data coming from the three units are collected either by means of an ANT USB2 Stick that is plugged into a personal computer during the acquisitions or by an Android smartphone that supports ANT.

The raw sensor data read by the IMU are composed of three accelerometer components, three gyroscope components and three magnetometer components and they are sent to the nRF52832 with a 40 Hz rate. The microcontroller, then, computes the 9-axis quaternion with the algorithm developed by Madgwick *et al*.,^28^ transmitting one quaternion out of four to the USB2 stick or smartphone, resulting in a 10 Hz frequency, through the ANT communication protocol.

**Figure 3:**
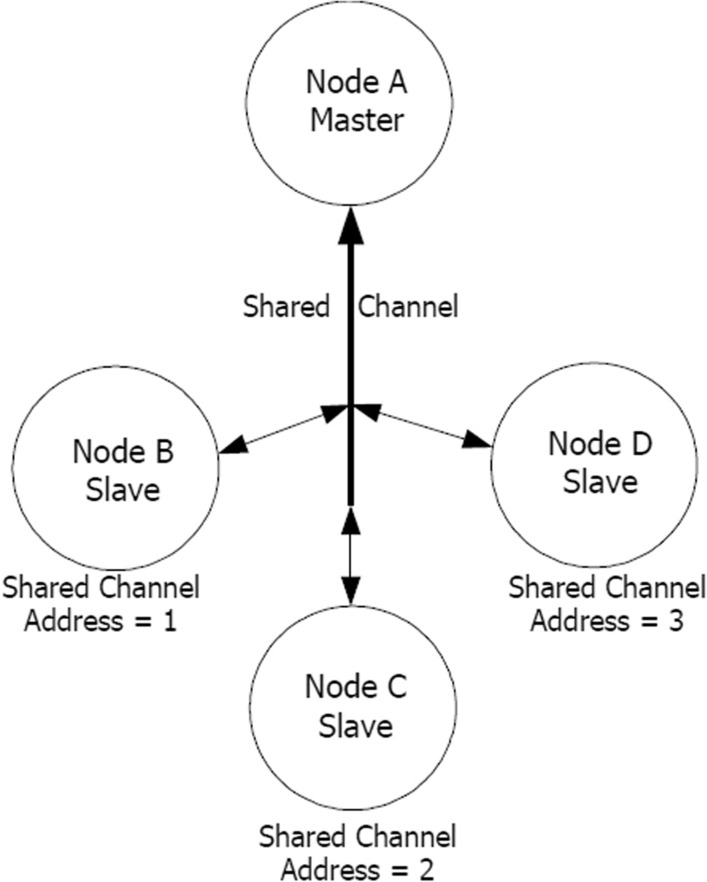
Schematic representation of the ANT Shared Channel topology implemented in this research work

The USB2 Stick or the smartphone is the receiver of the data sent by the nRF52832 and it is configured as the master, while the peripheral units work as the slaves. The topology of the network is called Shared Channel and is shown in Fig. 3. The master channel has a channel period of 30 Hz, so that it has a 10 Hz time slot to address each of the units.

Each sent quaternion is expressed through a floating-point value, ranging from − 1 to 1 and it is transmitted in a byte of the data payload. Moreover, it is also present a counter, increased every four quaternions calculated with a frequency of 40 Hz, to identify the n-th transmission.

### Respiratory Signal Processing

The process which leads to the respiratory parameters extraction from the data collected by the units is performed offline using a software that implements the previously validated algorithm by Cesareo *et al*.^17^ In the cited work, the estimation had a mean absolute error < 2 breaths/min with respect to a gold standard measurement (optoelectronic plethysmography) and was tested only in static conditions. The choice of the parameters corresponds to what was used in the previously validated algorithm in static conditions.

Preliminary validation data during dynamic activities (walking and running at different speeds) showed good agreement between the presented IMU-based system and a Cosmed K5 metabolic cart, as shown in a work by Angelucci *et al*.^7^ In dynamic conditions, a fine-tuning of the algorithm by Cesareo *et al*.^17^ to adapt the code to the processing of breathing during dynamic activities was added by including the knowledge of the performed activity in the respiratory signal processing.^6^ In particular, the variations are in the cut-off frequencies of some of the implemented filters. The whole elaboration algorithm can be subdivided into four main parts: pre-processing, dimension reduction, spectrum analysis, and processing.

**Figure 4:**
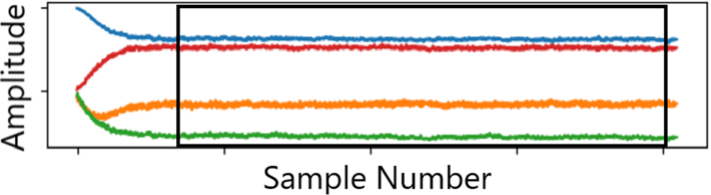
Window selection to remove the initial transient period at the beginning of each activity or posture.

In the pre-processing phase, the data divided by unit of origin are organized in four arrays, where missing data are replaced after interpolation is performed. The quaternions are created by combining the arrays. Then, the window to be analyzed is manually selected to avoid a transitory phase necessary for the quaternion to stabilize. The same selection was done in to train the HAR algorithm that is presented in the next section. An example of window selection of a single unit is shown in Fig. 4.

Afterwards, the quaternion product is computed, providing the orientations of the thoracic and abdominal unit referred to the orientation of the reference unit. (1) and (2) show how these computations are performed:

1 $${}_{{{\text{Ref}}}}^{{{\text{Th}}}} \hat{q} = {}_{{{\text{Earth}}}}^{{{\text{Th}}}} \hat{q} \otimes {}_{{{\text{Earth}}}}^{{{\text{Ref}}}} \hat{q}* = {}_{{{\text{Earth}}}}^{{{\text{Th}}}} \hat{q} \otimes {}_{{{\text{Ref}}}}^{{{\text{Earth}}}} \hat{q}$$

2 $${}_{{{\text{Ref}}}}^{{{\text{Ab}}}} \hat{q} = {}_{{{\text{Earth}}}}^{{{\text{Ab}}}} \hat{q} \otimes {}_{{{\text{Earth}}}}^{{{\text{Ref}}}} \hat{q} * = {}_{{{\text{Earth}}}}^{{{\text{Ab}}}} \hat{q} \otimes {}_{{{\text{Ref}}}}^{{{\text{Earth}}}} \hat{q}$$

‘Th’ indicates the thoracic unit, ‘Ab’ the abdominal unit, and ‘Ref’ the reference unit. ${}_{y}{}^{x}\widehat{q}$ is the quaternion describing the position of a generic point x with respect to a generic point y, and all quaternions are expressed in the same way. For instance, ${}_{{{\text{Ref}}}}^{{{\text{Th}}}} \hat{q}$ describes the position of the thoracic unit with respect to the reference unit. * refers to the quaternion conjugate, and ⊗ to the quaternion multiplication.

After the quaternion product, the non-respiratory movements are reduced because angular changes are referred to the reference unit, which does not detect breathing-related motions, but it is integral with trunk movement. Then, the baseline is computed by means of the moving average on 97 samples for each quaternion component and subtracted to them to remove the residual non-breathing movement. The generated components are the input for the dimension reduction block.

With the aim of reducing the dimension of the dataset, the Principal Component Analysis (PCA)^36,43^ is performed. The first component, the one with the greatest amount of variance explained, is computed for the thorax and for the abdomen and considered as respiratory signal and constitute the basis for the spectrum analysis.

The generated signals are filtered with a Savitzky-Golay FIR (Finite Impulse Response) smoothing filter of the 3^rd^ order with a window length of 31 samples. This filter works using the linear least squares method to fit successive sub-sets of adjacent data with a third order polynomial. In this way, the noise is decreased without changing the shape and the signal peaks height. Then, the mean (*f*_mean_) and the standard deviation (*f*_std_) of the inverse of the distances between subsequent peaks are considered to obtain a frequency estimate. *f*_mean_ and *f*_std_ are used to compute *f*_thresh_ and the procedure is performed both for the thoracic component and the abdominal component as in (3):

3 $$  f_{{{\text{thresh}}}}  = \max \left( {f_{{\text{thresh}}\_{\text{min}}} ,\,f_{{{\text{mean}}}}  - f_{{{\text{std}}}} } \right) $$

$f_{{\text{thresh}}\_{\text{min}}}$ is arbitrarily set at different values for static postures (0.05 Hz, as in Cesareo’s algorithm) or dynamic activities (0.2 Hz for walking and cycling, 0.4 Hz for running), so that a better filtering of low-frequency components of dynamic activities is guaranteed.

Once the threshold frequencies of both the thoracic and the abdominal unit are obtained, the low-frequency threshold is computed as the minimum between the abdominal low threshold and the thoracic low threshold. The use of a low threshold helps in the identification of the power spectral density (PSD) peak related to the RR and does not consider very low frequency peaks, often caused by movement artifacts.

Subsequently, the PSD estimate is computed employing the Welch's method, with the Hamming window type, 300 samples as window size, and 50 samples of overlap.

The PSD maximum in the interval between the computed low threshold and a maximum (1 Hz for static postures, 0.75 Hz for walking and cycling, 1.4 Hz for running) is identified (*f*_peak_). This value is used to build the adaptive band-pass filter settings (centered in *f*_peak_). The upper and lower cut-off frequencies, for both thorax and abdomen, are obtained as in (4) and (5):

4 $$f_{{\text{U}}} = f_{{\text{peak}}} + 0.04\, {\text{Hz}}$$

5 $$f_{{\text{L}}} = \max \left( {f_{{{\text{thresh}}_{{\text{m}}} }} ,\, f_{{\text{peak}}} - 0.04\, {\text{Hz}}} \right)$$

The final processing block comprises all the processes intended to extract breathing frequency and other respiratory parameters from the signals obtained after the dimension reduction block.

The first step is the application of the band-pass filter with the previously set cut-off frequencies ${f}_{\text{U}}$ and ${f}_{\text{L}}$. Since the frequencies are dependent on ${f}_{\text{peak}}$, the result is an adaptive filter, based on the specific analysed recording.

Then, a parametric tuning based on the ${f}_{\text{peak}}$ value is performed. This is necessary for the subsequent steps of filtering and maxima and minima detection. In particular, the involved parameters are the window length in terms of samples for the third order Savitzky–Golay filter and the minimum peak distance. In fact, the algorithm chooses the tallest peak in the signal and ignores all peaks within the decided distance and the minimum prominence threshold, through which is possible to set a measure of relative importance; a more detailed description of the parameters can be found in the work by Cesareo *et al*.^17^

Afterwards, filtered signals are furtherly smoothed through the application of a third order Savitzky–Golay FIR filter, to optimize subsequent detection of maxima and minima point, which are identified applying the parameters previously set. Moreover, in addition to the thoracic and the abdominal signal, the process is repeated also for the sum of the two signals once they were filtered with the Savitzky–Golay filter. RR is thus obtained breath-by-breath and the values obtained for each posture or activity are reported in Section "Results".

### Human Activity Recognition Algorithm

After the window selection, shown in Fig. 4, the data processing to train the activity recognition algorithm is different from the one for the respiratory analysis. All parameters of the algorithms are determined empirically to maximize the outcome.

The next step involves the creation of a single large dataset containing all the activity proper labeled for all the subjects. In a first phase, the algorithm is run on the data of the reference unit, because it can be considered representative of the subject’s positions. Secondly, the algorithm is trained on the signals coming from all three units (thoracic, abdomen, and reference). In both cases, a dataset is obtained merging the tasks “sitting without support” and “sitting with support” in a single label called "sitting", and the tasks “walking at 4 km/h” and “walking at 6 km/h” in a label called "walking", so the final dataset has 10 labels. This is done to increase the variability of the signal during the training process, so that the algorithm can be more robust in identifying a person that sits with a back support from a supine one and between a fast walk and a run.

The resulting dataset is unbalanced, because the labels "sitting" and "walking" had about twice the data of the other labels. The unbalancing is kept in the situation with one unit, while data are balanced in the training with three units. A balancing procedure is used, which consists in reducing the samples of each label to same number of the activity with the lowest amount of data.

The implemented preprocessing steps are data standardization, label encoding and segmentation. Standardization is performed with (6), so that data are centered on 0 and properly scaled. $\mu$ is the mean and $\sigma$ is the standard deviation.

6 $$x_{{{\text{std}}}} = \frac{x - \mu }{\sigma }$$

The data are then segmented in non-overlapping windows of 200 samples in length, equal to 20 s of recording.

After these steps, splitting into training and test sets is required. It is chosen to use 80% of the data for the training set and the remainder 20% for the test set. The seed to the random generator was set equal to 42.

In machine learning methods the feature extraction must be performed before the model training. The selected features are both in time domain and frequency domain for one unit, while only the ones in time domain were used for the three units.

The time domain features are extracted from the time series of the signal and are the following: mean, standard deviation, variance, kurtosis, skewness, peak-to-peak distance, median, interquartile range. The frequency domain features are extracted from the Fast Fourier Transform (FFT) of the signal and are the following: mean, standard deviation, skewness, maxima and minima of the FFT, mean and maximum of the power spectral density.

Three machine learning methods are used: a K-Nearest Neighbor classifier (KNN), a Random Forest classifier (RF) and a Support Vector Machine (SVM).

In the case of the KNN classifier, the metric chosen for the computation of the distance is the Euclidean metric.^34^ The optimal number of neighbors K is around 5, since afterwards the accuracy score decreased.

In the case of the RF classifier,^40^ the splitting rule to create the nodes of the trees that compose the forest is the Gini Criterion. Afterwards, in each node the corresponding attribute is chosen by minimizing the impurity, as it is traditionally done with RF classifiers.

Since the data of this research project cannot be separated linearly in the original space, to develop a SVM a kernel is used. In this case, the Radial Basis Function Kernel is used, which can be expressed mathematically as (7):

7 $$K\left( {X_{1} ,X_{2} } \right) = \exp \left( { - \frac{{\left\| {X_{1} - X_{2} } \right\|^{2} }}{{2\sigma ^{2} }}} \right)$$

where $\sigma$ is the variance and the hyperparameter $\Vert {X}_{1}-{X}_{2}\Vert$ is the Euclidean distance between two points ${X}_{1}$ and ${X}_{2}$. In this case, distance is used as an equivalent of dissimilarity: when the distance between the points increases, they are less similar. By default, $\sigma$ is taken equal to one, so the kernel is represented by a bell graph, that decreases exponentially as the distance increases and is 0 for distances greater than 4.

**Figure 5:**
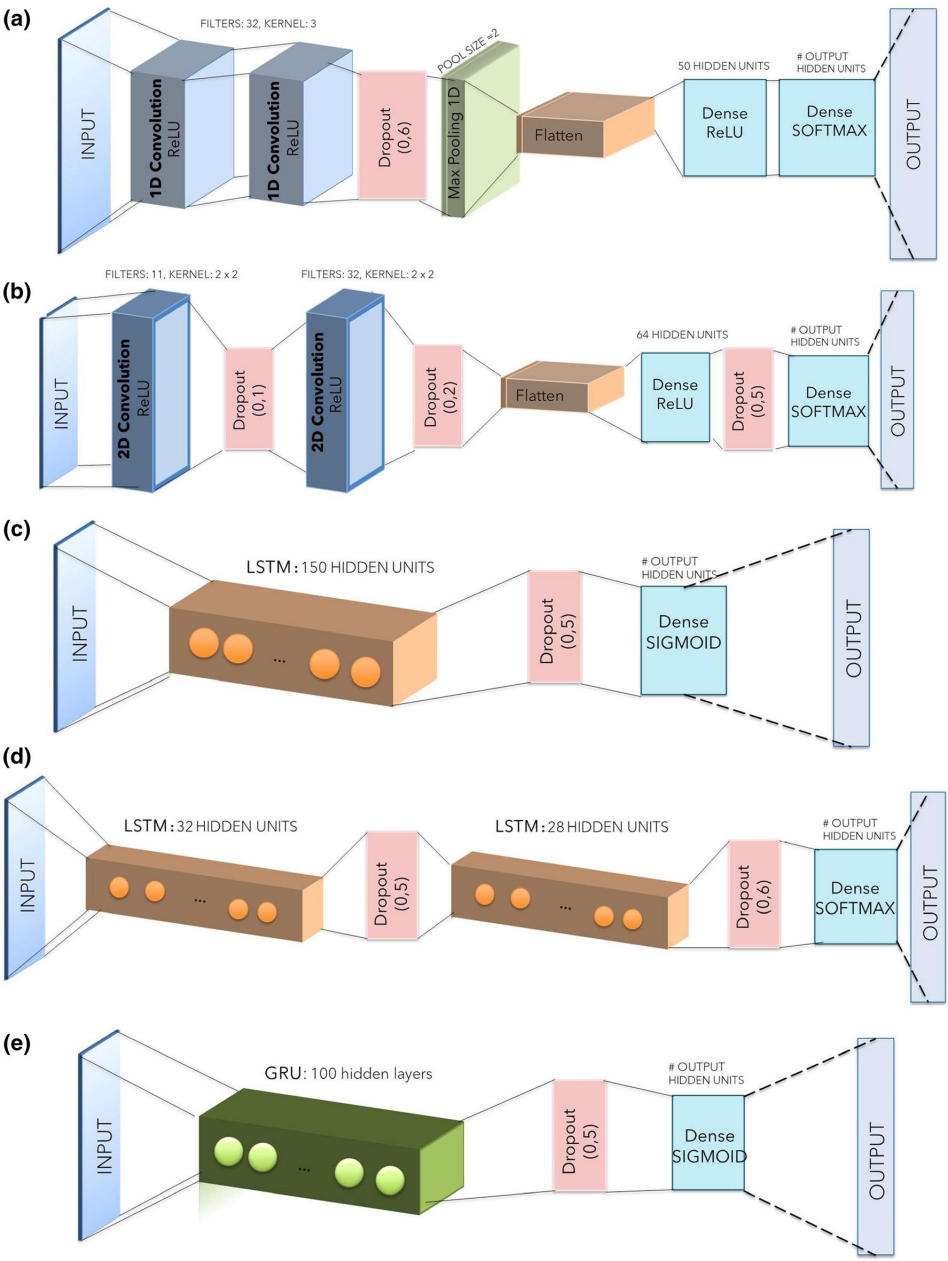
Deep Learning Networks implemented in this research project. (a) 1D Convolutional Neural Network (1DCNN); (b) 2D Convolutional Neural Network (2DCNN); (c) Single-layer Long Short Term Memory (BASE LSTM); (d) Multi-layer Long Short Term Memory (MULTI LSTM); (e) Gated Recurrent Network (GRU).

Five additional networks are created using deep learning methods; their characteristics are shown in detail in Fig. 5. The networks used are the following: a 1D Convolutional Neural Network (1DCNN), a 2D Convolutional Neural Network (2DCNN), a single-layer Long Short Term Memory (BASE LSTM), a multi-layer Long Short Term Memory (MULTI LSTM) and a Gated Recurrent Unit (GRU).

All networks use the Sequential Model to construct a plain stack of layers where each layer has exactly one input tensor and one output tensor. During the optimization part of the algorithm, the error at the current state must be iteratively estimated. For all networks, the chosen loss function is the Sparse Multiclass Cross-Entropy Loss as in (8), used to calculate the model’s loss such that the weights can be updated to minimize the loss on subsequent evaluations. The Cross-Entropy loss is defined as:

8 $$J\left( w \right) = - \mathop \sum \limits_{i = 1}^{N} y_{i} \log \left( {\hat{y}_{i} } \right)$$

where $w$ refers to the model parameters, ${y}_{i}$ is the true label and ${\widehat{y}}_{i}$ is the predicted label.

After that, to reduce the losses, an optimizer is used to adjust the neural network’s attributes such as weights and learning rate. The optimization method for the CNNs and GRU used is the Adam optimizer^25^ based on adaptive estimates of lower-order moments. For the LSTM networks, the RMSprop optimizer is used. The batch size is a hyperparameter that defines the number of samples taken from the training dataset to train the network before updating the internal model parameters; the chosen value is 16.

### Questionnaire

An evaluation questionnaire was given to the subjects participating in the study to collect impressions about usability, acceptance, and wearability of the wearable system. It is based on the System Usability Scale (SUS)^14^ and consists of 10 items, with odd-numbered items phrased positively and even-numbered items phrased negatively. The evaluation criteria of the SUS usability questionnaire were maintained in this application. In particular, the 10 items included in the ad-hoc questionnaire for the device evaluation are listed in Table 1.

**Table 1:** List of items in the ad-hoc questionnaire.

Item number	Description	Best score	Worst score
I1	I think that the device is easy to wear and place	5	1
I2	I think that I need support to handle the device	1	5
I3	The attachment method of the device units makes the placement easier and improves wearability	5	1
I4	I would have preferred to remove the device during some activities	1	5
I5	I think I would be able to use the device independently	5	1
I6	I found the attachment method uncomfortable	1	5
I7	I think I could wear the device for a long time	5	1
I8	I think that using the device would negatively affect my daily life activities	1	5
I9	I think I could sleep as usual while wearing the device	5	1
I10	I had irritation and/or itching in the area of attachment during the test	1	5

The items are presented as 5-point scales numbered from 1 (“Strongly disagree”) to 5 (“Strongly agree”) and the subject had to give a score to each item. Age, gender, weight, and height were asked at the beginning of the questionnaire.

## Results

### Respiratory Rate

**Figure 6:**
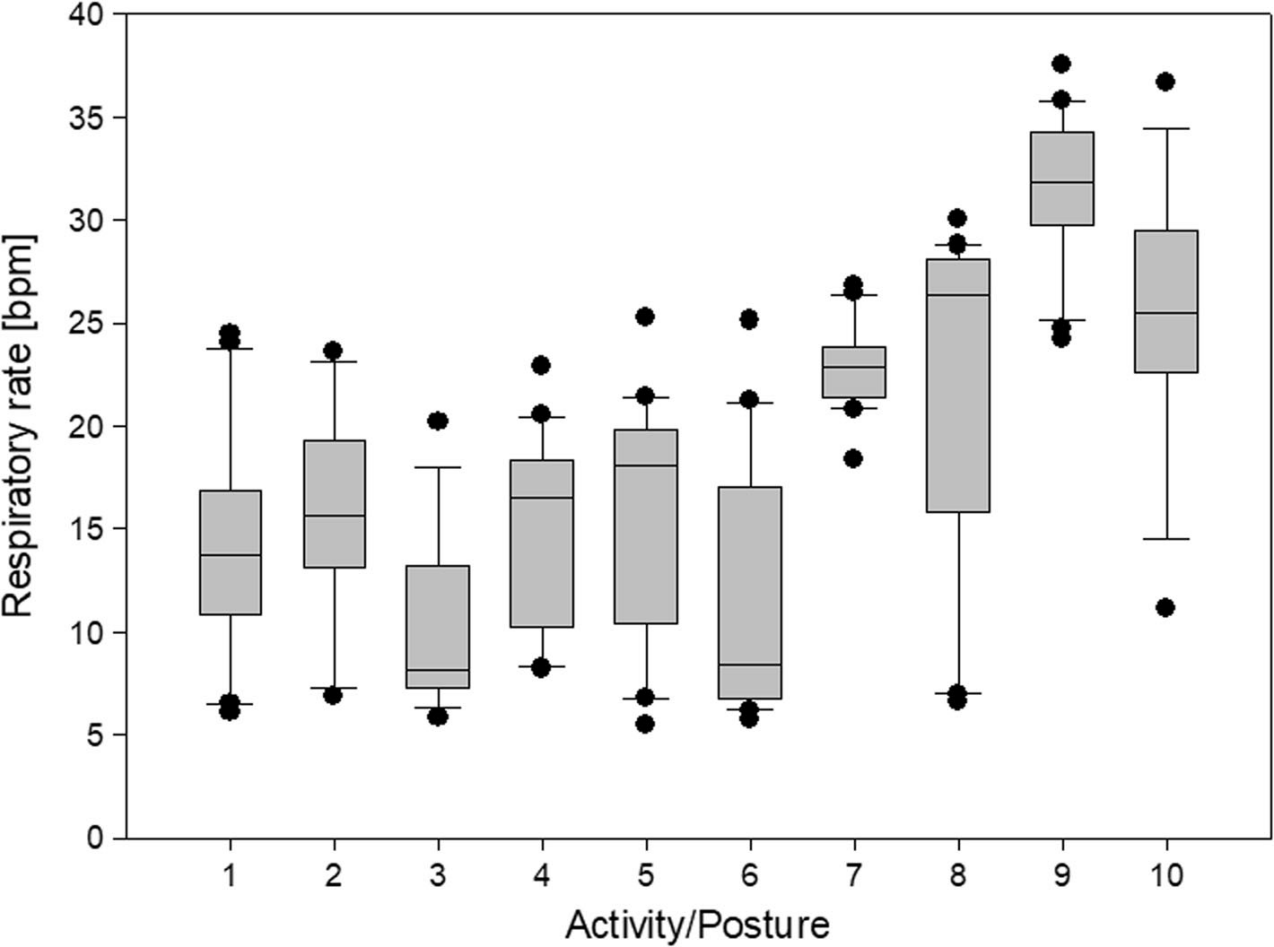
Boxplots of the obtained values of RR in the different postures and activities. The labels are the following: 1—Sitting; 2—Supine; 3—Prone; 4—Lying Left; 5—Lying Right; 6—Standing; 7—Walking slow; 8—Walking fast; 9—Running; 10—Cycling.

RR was studied for the 20 involved subjects in the different postures and activities. Due to the unfavorable signal-to-noise ratio, parameters could not be extracted in the case of climbing stairs with the previously validated algorithm, therefore those values are not included in the analysis. The dataset presented puts together the two sitting positions but separates “walking slow” and “walking fast” to show the sensitivity of the respiratory analysis algorithm to the different levels of effort. The boxplots of the median values obtained for each subject in the different conditions are shown in Fig. 6.

The distributions were statistically compared one with the other with a non-parametric Friedman test. The Shapiro–Wilk normality test was failed (*p* < 0.05); also, the Equal Variance Test (Brown-Forsythe) was failed (*p* < 0.05). The differences in the mean values among the groups were greater than would be expected by chance; there is a statistically significant difference (*p* ≤ 0.001).

To isolate the group or groups that differ from the other, the Bonferroni t-test was used as multiple comparison procedure. The p-values obtained with these comparisons were analyzed. The activities, “walking slow”, “walking fast”, “running” and “cycling” have a statistically significant difference with respect to static postures (*p* < 0.05 in all cases), but not always one with respect to the other. “Walking slow” and “walking fast” do not significantly differ from “cycling” (*p* = 1.000) and between one another (*p* = 1.000). This result confirms what is known in the literature,^9,10^ i.e. that during physical activity RR increases and this phenomenon is more evident when the activity is more demanding (during “running”). Also, there is a statistically significant difference between the “supine” and the “prone” positions (*p* = 0.045) and between the “lying right” and the “prone” positions (*p* = 0.049). This is likely due to the fact that the processing algorithm is designed to analyze the movement of the two units in the front with respect to the reference unit, while in prone position also the dorsal movement contributes to ventilation.^3^

### Human Activity Recognition Algorithm

The results obtained with the previously introduced methods with one unit are reported in Table 2.

**Table 2:** Evaluation metrics of the algorithms using data from the reference unit.

Method	Accuracy	Precision	Recall	F1-score
KNN	0.92	0.94	0.95	0.94
RF	0.96	0.96	0.95	0.95
SVM	0.91	0.92	0.91	0.92
1D CNN	0.96	0.97	0.96	0.96
2D CNN	0.92	0.90	0.88	0.88
BASE LSTM	0.91	0.95	0.94	0.95
MULTI LSTM	0.90	0.88	0.88	0.88
GRU	0.97	0.97	0.97	0.97

Looking at the accuracy, the best performing model is the GRU.

The results obtained with the previously introduced methods with three units are reported in Table 3. From the confusion matrix that can be obtained considering every individual position or activity, the highest confusion happens when the sitting position is predicted as supine, and vice versa. This is observed both in case with one unit and in the case with three units. This is probably because the posture 'sitting' includes data obtained when the subjects were sitting with and without a back support.

**Table 3:** Evaluation metrics of the algorithms using data from the three units.

Method	Accuracy	Precision	Recall	F1-score
1D CNN	0.99	0.99	0.99	0.99
2D CNN	0.99	0.99	0.99	0.99
BASE LSTM	0.97	0.97	0.97	0.97
MULTI LSTM	0.98	0.98	0.98	0.98
GRU	0.98	0.98	0.98	0.98
KNN	0.98	0.98	0.98	0.98
RF	0.99	0.98	0.98	0.98
SVM	0.98	0.98	0.98	0.98

**Figure 7:**
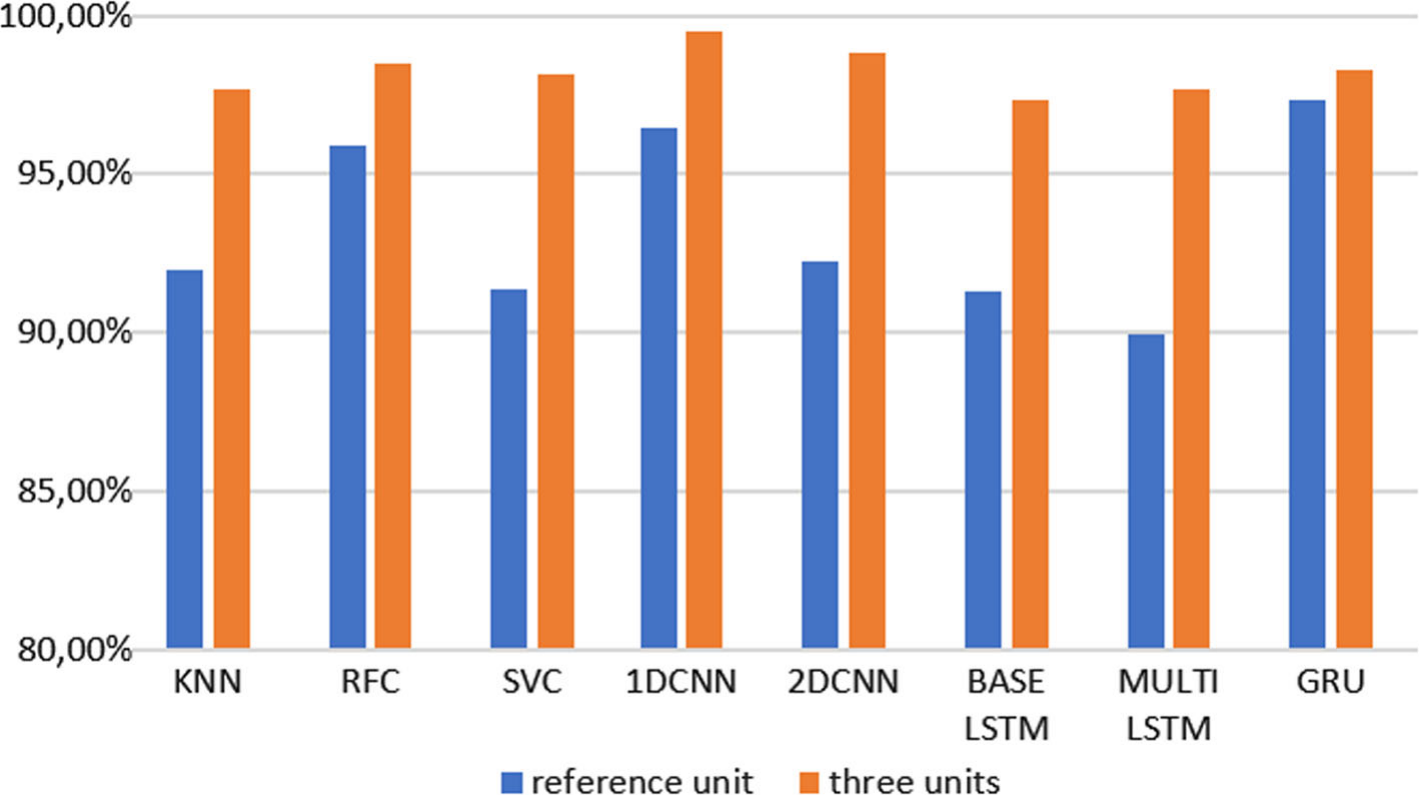
Comparison between the accuracy of the different machine and deep learning methods with one unit (*i.e.*, the reference unit) and three units.

A direct comparison between the HAR methods developed with one unit and those developed with three is shown in Fig. 7.

In this case, all methods have better performances when compared to the case with only one unit. The best performing one is the 1DCNN. It must be considered also that the features extracted for the three units are only concerning time, which suggests that the inclusion of the frequency features could further improve the accuracy.

### Questionnaire

**Figure 8:**
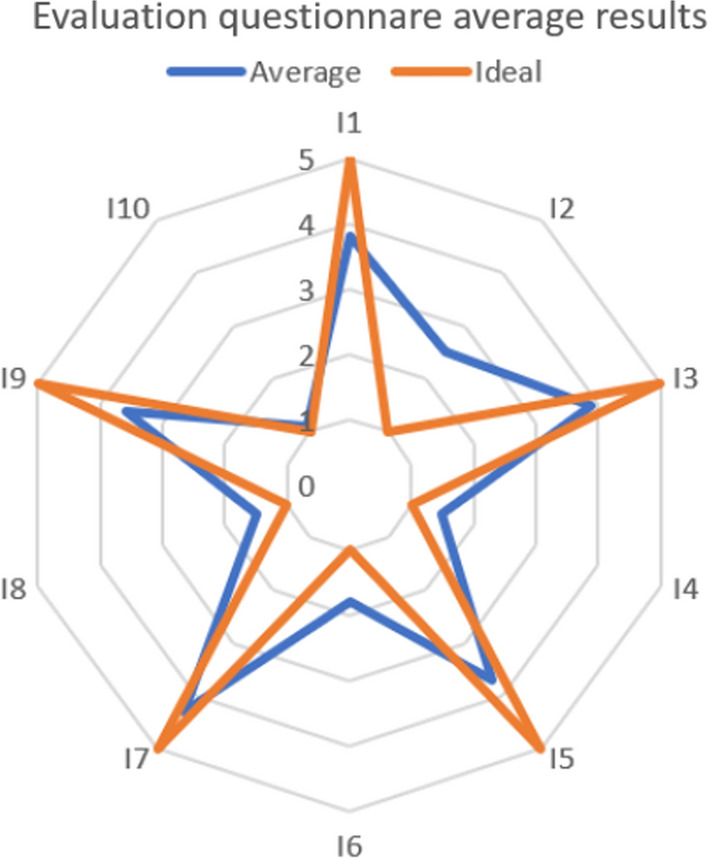
Radar chart of the evaluation questionnaire average results compared to the ideal score.

The subjects found the device easy to wear and place, however most claimed they needed the support to manage the device. Nearly eighty percent asserted that the device attaching method makes placement easier and improves wearability. Moreover, nobody reported irritation or itching in the device mounting areas, and almost no one wanted to take their device off during activities. Around 70% of the participants believe the device would not interfere with their daily activities and that they would be able to sleep while wearing it. Almost everyone believes that it is possible to keep the device for long time, but only a few say they can use it independently, probably because the reference unit is placed on the lower back, so another person is needed to place the unit properly at the beginning of each acquisition. The actual results are shown in Fig. 8 in comparison to the ideal ones.

## Discussion

In the presented sensor system,^15,16^ two sensor units are aimed at detecting chest wall breathing-related movements, one is located on the thorax and the other on the abdomen; the last IMU is placed on a position not involved in respiratory motion but integral with body movement, most often on the lower back. In fact, the influence of posture on chest wall motion is intensively studied in the literature. It was previously studied that most of the chest wall volume change is distributed in the thoracic compartment in vertical postures and in abdominal compartment in horizontal postures.^37^ For this reason, the system’s configuration is particularly advantageous for a thorough analysis of the chest movement during breathing in different postures since it can account for the different contributions of the chest wall in different positions.

The algorithm to extract breathing parameters allowed to obtain results that confirm what is known in the literature,^9,35^ *i.e.*, that the frequency is higher in dynamic conditions, and increases for increasing efforts. Furthermore, it must be noted that most systems are not able to provide measurements of RR during demanding dynamic activities like running.

With a previous knowledge of the performed activity, the respiratory signal processing can be fine-tuned, and this system is able to accurately measure this signal even during dynamic activity. A future improvement of the project includes a new artificial intelligence algorithm that combines HAR to automatically decide how to process respiratory data.

A drawback of the actual signal processing algorithm is that it requires a manual analysis window selection, but this can be easily overcome by implementing a sliding window algorithm.

This system is advantageous both for sports and medical applications, due to its ability to measure this parameter in a broad range of situations. However, the signal-to-noise ratio is too low while climbing the stairs and the algorithm could not be applied.

The results obtained showed an overall good capability to recognize different activities, independently from the age or the gender of the subjects. Although only features in time and not in frequency were used in the case with three units, the comparison between the use of a single units compared to the use of three, showed that the second case works better, with higher accuracy and f1-score both for machine and deep learning methods. The dataset is however small and during dynamic activities with a prominent frequency component, like running or walking at a fixed speed. Data should be collected from more subjects and in more diverse condition to make the algorithm robust for use outside of laboratory settings. A final consideration regards the first step of the data preparation: removing the initial transitory might have led to an overestimation of the accuracy.

This work can be considered original with respect to state of art of HAR because there are many studies that works with the output data of the accelerometers and gyroscopes, while there are few that use only quaternion data. The three units only send quaternion data, which allows to reduce the dimension of the dataset (4 measures instead of 9 of the IMU). Only using the quaternion allows to send all the needed data in a single package without losing information. Quaternions allow to distinguish quite well between activity orientation-related, such as lying left and lying right or supine and prone. It is also worth noting that the device combines breath analysis with activity recognition, making it very innovative.

A limitation of this work is that the results of respiratory parameters were not compared with a gold standard, so accuracy of the adapted algorithm cannot be assessed. However, the technical feasibility of this solution was demonstrated. A trial on healthy subjects during dynamic activities is needed to fully validate the algorithm for dynamic activities. Subsequently, a trial on patients can provide physiological results that are clinically significant and that can be effectively used for telemedicine purposes without the supervision of the clinician. The proposed device could be applied in the case of chronic respiratory diseases, such as COPD or asthma, but also in cardiac diseases in which RR is predictive of an adverse event, such as heart failure or cardiac arrest. It is also possible to test this solution to monitor at home the acute phase, the rehabilitation phase, and the long-term clinical outcomes of Covid-19. A feature that is needed for patient monitoring is the generation of real-time alerts, but to determine what thresholds or trends constitute a critical event a pilot study is needed to gain relevant clinical data.
